# Comorbidities and Ethnocultural Factors Limit the Physical Activity of Rural Sri Lankan Patients with Diabetes Mellitus

**DOI:** 10.1155/2018/4319604

**Published:** 2018-03-06

**Authors:** Arjuna Medagama, Manoj Galgomuwa

**Affiliations:** Department of Medicine, University of Peradeniya, Peradeniya, Sri Lanka

## Abstract

South Asians have high prevalence of diabetes, cardiovascular risk, and physical inactivity. Reasons for physical inactivity have not been explored among Asians living within their endogenous environments. During phase 1 of the study, we assessed the physical activity (PA) of the population using a quantitative, descriptive, cross-sectional research method. During phase 2 of the study, a qualitative method with in-depth interviews was used to collect data on barriers of PA. Four hundred patients with type 2 diabetes, comprising 113 (28.2%) males and 287 (71.7%) females, were enrolled. The overall prevalence of physical inactivity was 21.5% (males: 15.9%, females: 23.7%). The majority (44.8%) of the study population was active and 33.8% were minimally active. The mean weekly MET minutes was 4381.6 (SD 4962). The qualitative study (*n* = 45) identified health-related issues—lifestyle and time management and social embarrassment, prioritizing household activities over PA as significant factors that limited PA.

## 1. Background

Sri Lanka is a developing country with a population of 21 million inhabitants and a rapidly increasing burden of noncommunicable diseases (NCD) [1].

In 2005, the prevalence of hypertension, diabetes, and dysglycaemia was 20%, 11%, and 20%, respectively [2]. The mortality from cardiovascular disease in Sri Lanka is recognized to be one of the highest worldwide [3]. The exact reason for this high mortality is unknown but is probably contributed by the clustering of both modifiable and nonmodifiable cardiovascular risk factors and the genetic variability that is due to differences among populations [4, 5].

Physical inactivity is identified as the fourth leading risk factor for overall mortality globally and one that is readily modifiable [6, 7]. Sedentary living is responsible for one-third of deaths due to coronary heart disease (CHD) and diabetes, diseases for which physical inactivity is a risk factor [8].

Diet and PA are 2 key modifiable risk factors that play an important role in the incidence, management, and outcomes of diabetes [9]. The American Diabetes Association (ADA) recommends 2 types of PA for individuals with diabetes, which includes aerobic exercises and strengthening exercises. The ADA recommends 30 minutes of moderate- to vigorous-intensity aerobic exercise for at least 5 days a week or up to a total of 150 minutes per week and strength training of some type at least two times per week in addition to aerobic activity [10]. PA is recognized to increase insulin sensitivity, reduce cardiovascular risk factors, reduce mortality, and improve quality of life [11].

Very few studies have been performed in Sri Lanka that attempted to quantify PA among adults. Katulanda et al. performing the first national survey reported that over 60% of all Sri Lankan adults to be physically “highly active” [7]. Limited data is available on the PA patterns of Sri Lankan adults with diabetes. A subset analysis of the above study revealed that 13.9% of patients with diabetes are inactive.

However, the reason leading to “physical inactivity” has not been adequately explored in Sri Lanka previously.

The aim of the current study was to quantify physical activity and to find reasons for physical inactivity among a group of patients with type 2 diabetes, attending a large tertiary care diabetes facility in Sri Lanka. The study also attempted to explore the relationship of physical inactivity to sociodemographic, clinical, and biochemical parameters among the participants.

## 2. Methods

Institutional ethical clearance was obtained from the Institutional Ethics Review Committee (IERC) of the Faculty of Medicine, University of Peradeniya, Sri Lanka. All participants gave informed written consent. The study was performed at the diabetes facility at the Teaching Hospital, Peradeniya, Sri Lanka, from the 2nd of February 2015 to the 26th of August 2015.

The Teaching Hospital, Peradeniya, located on the outskirts of a major city, serves a catchment population of semiurban and rural dwellers.

During phase 1 of the study, we assessed the PA of the population by using a quantitative, descriptive, cross-sectional research method. During phase 2 of the study, a qualitative approach of semistructured questions with in-depth interviews to collect data on barriers of PA and factors that would facilitate PA was utilised.

Having a convenient sample of 400, consecutive patients with type 2 diabetes fulfilling the inclusion criteria were recruited for the first phase of the study.

Inclusion criteria were patients with type 2 diabetes mellitus diagnosed according to prevalent ADA criteria and age between 19 to 70 years. Pregnant and lactating females were not included in the study.

The study tool used to quantify PA was the interviewer-administered short form of the International Physical Activity Questionnaire (IPAQ) [12] in English and a validated version of the native language (Sinhalese) [13]. The short-form IPAQ allows continuous and categorical measurement of PA. The categorical score allows to estimate the time spent on light, moderate, and vigorous activities in a given week. The IPAQ scoring protocol assigns the following metabolic equivalent energy expenditure values to light-, moderate-, and vigorous-intensity activities: 3.3 METs, 4.0 METs, and 8.0 METs, respectively. The continuous score allows the estimation of the weekly energy expenditure expressed in MET minutes/week (metabolic equivalent task minutes). This is obtained by multiplying the value of energy expenditure for the given PA in MET by the weekly frequency (days per week) and the time in minutes (minutes per day). All questions on PA referred to the week immediately preceding the administration.

For the second phase, 45 participants were selected from phase 1 population using a structured stratified sampling method. The 45 patients thus selected were approached by a trained research assistant (RA) on their regular clinic day and were given an information sheet and verbal clarification regarding the study. Male and female patients who were between 19 and 70 years were recruited, and pregnant females were excluded from the study. Patients consenting to be included in the study were then given a date for an interview which was typically within 2 months of the initial recruitment date. In-depth interviews were selected over focus group discussions in this community as inhabitants of rural areas, and those with lower levels of education were believed not to communicate effectively within a group.

The clinical team looking after the patient was intentionally kept out of the data collection, as this may have influenced the answers provided by the subjects.

Each scheduled interview lasted an average 30 minutes and was undertaken at the Diabetes Clinic at the Teaching Hospital, Peradeniya. All interviews were conducted by one RA using a topic guide, which asked about the patients' reasons for not engaging in PA and barriers to initiating and maintaining an exercise schedule. The interviews were conducted using open-ended, semistructured questions to guide the participants and to maintain uniformity between all the interviews. The language of the interviews was Sinhalese as all the participants were fluent in this language. All patients were informed that their interviews will be recorded, transcribed, and analyzed while maintaining confidentiality.

The following sociodemographic data were obtained from the patients at the beginning of the interview and included information on the age, gender, marital status, occupation, income, area of residence, duration of diabetes, and other diabetes-related data.

## 3. Analysis

### 3.1. Phase 1: Quantitative Study

Data was analyzed using SPSS version 20. Continuous variables are presented in means (standard deviation) or median (interquartile range), when skewed in distribution. Categorical variables are presented as proportions or percentages. Comparisons of exercise-related parameters were made via analysis for variance for continuous variables and chi-square for categorical variables. A two-sided *P* value < 0.05 was defined as statistically significant. Bivariate and multivariate logistic regression analyses were used to determine associations between socioeconomic and health characteristics and physical inactivity. Pearson's χ^2^ was used to compare bivariate associations between socioeconomic and health variables and physical inactivity.

### 3.2. Phase 2: Qualitative Study

The interviews were digitally recorded and transcribed. The data were initially transcribed in Sinhalese, which was the native language of all the participants. Subsequently, 2 independent individuals fluent in both Sinhalese and English translated the transcribed data to English. The independent translations were compared with the research team with the help of the independent translators for accuracy of content. A final document was then prepared after consensus had been reached on the translated transcript contents. Data were analyzed using a framework approach. This involved the researchers reading through the transcripts and developing a matrix of overarching and supporting themes.

## 4. Results

### 4.1. Sociodemographic Characteristics

We recruited 400 patients with type 2 diabetes, comprising 113 (28.2%) males and 287 (71.7%) females. The mean age of the population was 55.4 (SD 8.9) years. The males were slightly older than the females. The patients were generally from a poor socioeconomic background with low levels of education, high unemployment, and low incomes. The mean BMI was 25.8 kg/m^2^. The females had higher BMI than the males (<0.001). The sociodemographic profile is given in Table 1.

The mean duration of diabetes in the study population was 8.5 (SD 6.8) years. Over half the population (51.5%) had at least one diabetes-related complication.

### 4.2. Quantitative Study: Physical Activity

The overall prevalence of physical inactivity was 21.5% (males: 15.9%, females: 23.7%). The majority (44.8%) of the study population was active and 33.8% were minimally active. The mean weekly MET minutes was 4381.6 (SD 4962). The males had a significantly higher value (5290) compared to females (4023, *P* = 0.02). The active group had a mean weekly MET minute of 8312 (SD 4908), followed by 1613 (SD 774.8) in the minimally active group and 275 (SD 206.8) in the inactive group. The mean weekly MET minutes are given in Table 2.

The waist circumference showed a negative correlation with mean weekly MET minutes (*P* = 0.03). A reduction in the mean weekly MET minutes was noted with advancing age and higher levels of income and education but did not reach statistical significance. Bivariate correlational studies showed that the mean weekly MET minutes were associated with gender (*P* = 0.02), but not with the duration of diabetes, presence of complications, weight, BMI, glycaemic control, or blood pressure.

Glycaemic control was not different between the active and inactive groups (active HbA1c 8.2% versus inactive HbA1c 7.9%, *P* = 0.74).

### 4.3. Qualitative Study

#### 4.3.1. Theme 1: Barriers to Physical Activity

Most participants (*n* = 38) mentioned that they encountered some form of barrier in engaging in regular PA. Three superordinate and 9 subordinate themes were developed following analysis with selected quotations from the transcripts. The barriers described by the participants were grouped into 3 themes; health related, time, and lifestyle management and social circumstances. More than half the participants had more than one barrier in engaging in exercise.

#### 4.3.2. Health Related

A large number (*n* = 18) of participants said joint-related problems prevented them from engaging in PA. The loose term “arthritis” was used to describe weight-bearing large joint problems. “Breathing problems” was described as another reason for not engaging in PA. Few (*n* = 3) described “chest pain when exercising” as a barrier.

#### 4.3.3. Time and Lifestyle Management

“Lack of time,” “inability to effectively manage time,” and “lack of motivation” was highlighted by both males and females as barriers to engaging in an exercise programme. Few (*n* = 15) described their daily chores, household activities, and employment as barriers in engaging in PA.

#### 4.3.4. Social and Cultural

The females said they were embarrassed and “uncomfortable” to engage in exercise in public areas. Most participants stated that they did not have access to a suitable facility for exercising. A majority stated that exercising outside their daily activities was alien to their lifestyle. Few males and females were concerned on how exercise performed within the home or their closed community would be accepted among others.

#### 4.3.5. Theme 2: Overcoming Barriers to Exercise

Availability of privacy to engage in exercise was felt to be essential among most females. Both males and females felt that the availability of equipment and dedicated areas for exercise would improve participation in PA. Most females felt that more support from family members to relieve them of their busy household schedules would encourage PA.

## 5. Discussion

In the current study, one-fifth of the study population was physically inactive. The study population had a high BMI and waist circumference. The population was middle aged to elderly, predominantly retired or unemployed, with low income and mostly educated up to the secondary school level. The sample was from a centre having a catchment of semiurban and rural areas.

Previous studies that quantified PA in the Sri Lankan population in general and among individuals with diabetes are few [7, 14]. The association of PA with socioeconomic parameters and health- and diabetes-related parameters has not been studied in the Sri Lankan population.

In the current study, the weekly mean MET minutes was relatively high compared to previous studies. Ranasinghe et al., studying a subset of patients with diabetes in Sri Lanka, reported a mean weekly MET minute of 3136 (SD 2059) [14]. The higher value of 4381 (SD 4962) in our study is probably explained by the sample being predominantly from a rural/semi urban community where PA from day to day chores and travel-related activity increases PA.

Although the self-reported rate of employment in our study was low, most rural inhabitants would engage in household-related farming and gardening, which may account for the higher MET minute values. This phenomenon has previously been reported in Sri Lanka [15]. Additionally, in rural Sri Lanka, transport-related PA is high due to lack of personal and public transport facilities resulting in most commuting on foot.

However, overall physical inactivity in the current study was 21.5% compared to the previously reported value of 13.9% among individuals with diabetes, 11% and 31.8% among the general adult population [7, 13]. These studies used the IPAQ for quantification of PA and are thus comparable.

We demonstrated a nonsignificant decline in PA with higher levels of income and higher education levels. This probably reflects the decline in PA in a rural population as people move away from manual labour to more sedentary jobs. Walking on foot may also be less due to wider access to other forms of personal transport such as motorcycles and cars with increasing income levels. Unfortunately, this cannot be assessed in the IPAQ, as this tool does not assess different domains of PA [16].

We found a significant association between physical inactivity and waist circumference but not with other health-related indices such as BMI, glycaemic control, and blood pressure.

Almost all the participants of the qualitative study said that they had barriers preventing them from engaging in PA. “Joint-related issues” and “breathing problems” emerged as limiting health issues. “Inability to prioritize time,” “lack of motivation,” and “inability to find time for PA” as a result of household chores or employment” were the commonest time- and lifestyle-related causes. Socially, embarrassment, prioritizing domestic activities over PA, and uncertainty on the social acceptance of exercising in public were common reasons for not engaging in PA.

Fear of engaging in PA in the presence of diabetes and other comorbidities has previously been reported in Sri Lanka and elsewhere [17–19]. Similarly, lack of motivation and inability to prioritize time have been reported as significant barriers to regular exercise in previous studies [20, 21]. The female participants in particular noted that they would be too embarrassed to undertake exercising in public areas as they felt this was culturally alien to them. This finding is in alignment with previously reported research performed in Asian ethnic minorities living in the UK and the USA [22–24].

Similarly, family and social pressures to prioritize domestic responsibilities over exercising among women of Asian origin has been previously reported as a barrier to PA [19, 22, 25].

The current study is unique that it was performed on a population of rural, low-income, and predominantly middle-aged to elderly population from a low resource setting. Previous qualitative studies on Asians and PA have originated from ethnic minority communities living in affluent societies [22, 23, 25, 26]. A recent meta-analysis has also confirmed the lack of data on PA, of patients living in their indigenous settings [26].

Although the mean weekly MET minutes observed in this study was higher than that reported previously, a larger proportion of subjects were physically inactive. Considering the benefits of PA in diabetes appropriate measures should be taken to encourage PA among patients with diabetes.

We recommend that patient education regarding PA be more specific leaving little or no ambiguity regarding the type and duration of PA. More robust and culturally accepted methods of knowledge transfer should be explored, such as the existing primary health care system. At the same time, PA prescription should be more individualized taking into account the physical disabilities and perceived negative outcomes and fears. Cultural and social beliefs and traditions should be taken into account in the formulation of PA activity guidelines at community and national levels. Development of infrastructure for PA should be aligned with cultural beliefs and social norms, in an attempt to incorporate PA into daily lifestyles such as walking. Empowering of rural communities and community leaders with the knowledge and benefits of PA may help it to be more widely accepted among the elderly, less educated, and the female-gender individuals.

As a limitation, the rural background, lower levels of education, and high-unemployment status may make the findings not to be nationally representative.

## 6. Conclusions

This study demonstrated that one-fifth of adult patients with diabetes are physically inactive and that more females are inactive than males. Similarly, the qualitative study demonstrated that most individuals had “barriers” that prevented them from effectively engaging in PA. Culturally, appropriate measures that take into account patients' perception of PA need to be urgently initiated.

## Figures and Tables

**Table 1:** Sociodemographic variables of the sample.

Variables	Male	Female	Total
(1) Gender	113 (28.25%)	287 (71.75%)	400
(2) Age (years)	56.4 (SD9.4)	55.03 (SD 8.7)	55.4 (SD 8.9)
(3) Education			
Primary education only	1 (0.25%)	10 (2.5%)	11 (2.75%)
Up to O/L	44 (11%)	113 (28.25%)	157 (39.25%)
O/L completed	37 (9.25%)	91 (22.75%)	128 (32%)
A/L completed	28 (7%)	71 (17.75%)	99 (24.75%)
Graduate/postgraduate	3 (0.75%)	2 (0.5%)	5 (1.25%)
(4) Occupation			
Unemployed	13 (3.25%)	202 (50.5%)	215 (53.75%)
Retired	37 (9.25%)	28 (7%)	65 (16.25%)
Employed	63 (15.75%)	57 (14.25%)	120 (30%)
(5) Income: LKR (USD)/month			
<10,000.00 (65.7)			225
10,000–30,000 (65.7–197)			144
30,000–50,000 (197–328)			23
>50,000 (>328)			8
(6) Duration of DM (years)	9.01 (SD 7.3)	8.3 (SD 6.6)	8.5 (SD 6.8)
(7) BMI (kg/M2)	24.7 (SD 3.4)	26.3 (SD 4.2)	25.8 (SD 4.1)
(8) Waist circumference (cm)	88.5 (SD 11.7)	93.5 (SD 10.1)	92.1 (SD 10.8)
(9) HbA1c %, (mmol/mol)	8.2 (SD1.9), (65)	8.1 (SD 1.8), (66)	8.2 (SD 1.86), (65)

**Table 2:** Physical activity levels among males and females.

Physical activity	Males	Females	*P*	Total population
(1) Vigorous activity mean MET minutes/week (SD)	2270 (4490)	840 (2447)	*P* = 0.02	1245 (3221)
(2) Moderate activity mean MET minutes/week (SD)	1290 (1650)	1626 (2116)	*P* = 0.07	1531 (2000)
(3) Walking mean MET minutes/week (SD)	1729 (2238)	1556 (2133)	*P* = 0.4	1605 (2161)
(4) Total mean weekly MET minutes/week (SD)	5290 (5532)	4023 (4682)	*P* = 0.03	4381 (4962)
(5) Active group: 179 (45%) Males: 59 (15%) Females: 120 (30%)	8832 (5538)	8133 (4718)	*P* = 0.7	8482 (4998)
(6) Minimally active: 135 (34%) Males: 36 (9%) Females: 99 (25%)	1982 (1357)	1629 (758)	*P* = 0.06	1723 (962)
(7) Inactive: 86 (21%) Males: 18 (5%) Females: 68 (17%)	297 (245)	256 (192)	*P* = 0.4	265 (204)

## Data Availability

Data is available with the authors.

## Abbreviations

PA: Physical activity
NCD: Noncommunicable diseases
ADA: American Diabetes Association.
